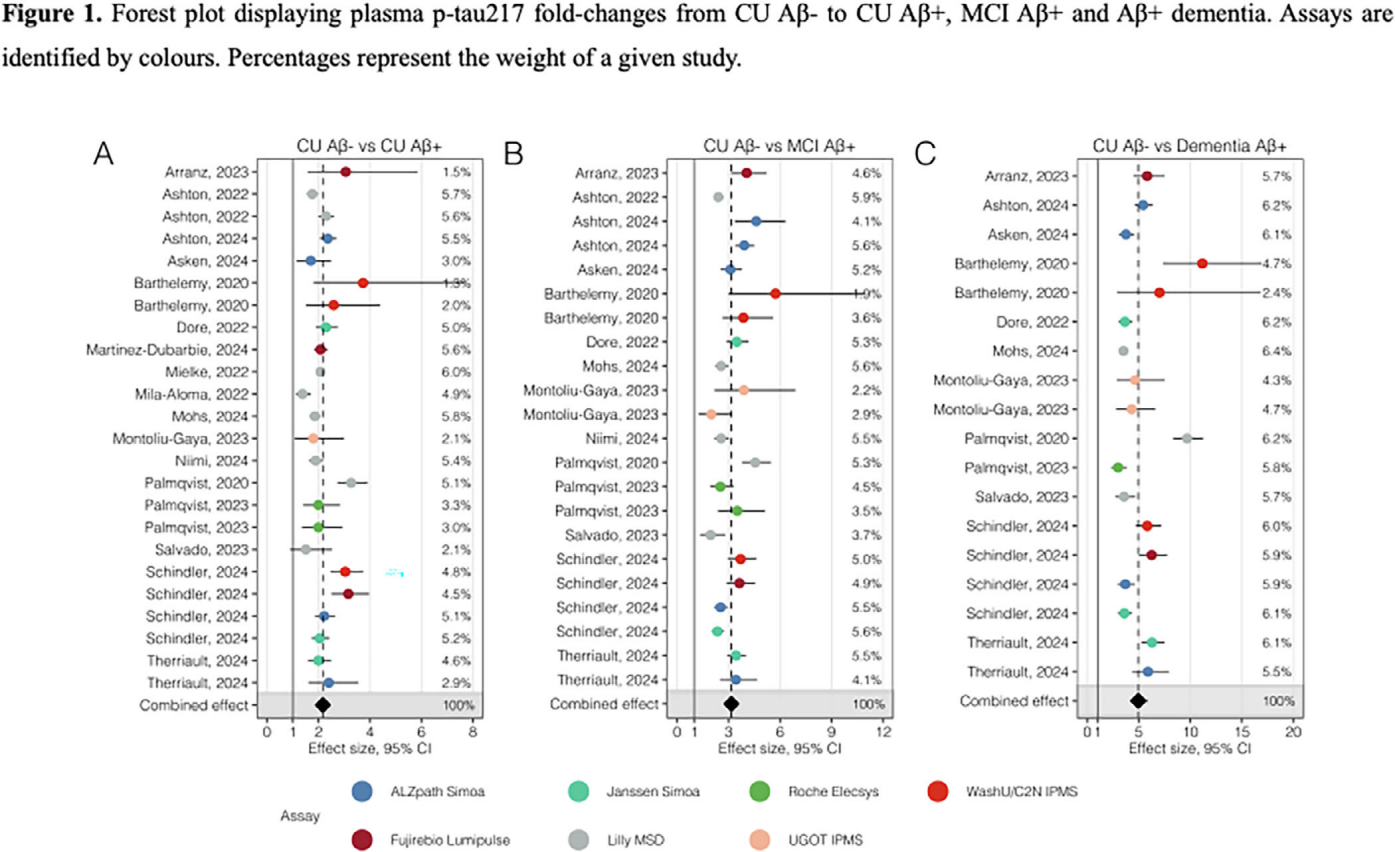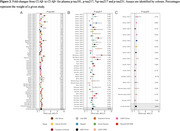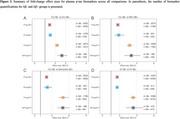# Plasma *p*‐tau changes across the clinical spectrum of Alzheimer's disease: a systematic review and meta‐analysis

**DOI:** 10.1002/alz70856_106492

**Published:** 2026-01-08

**Authors:** Wagner S. Brum, Joseph Therriault, Arthur C. Macedo, Lydia Trudel, Fernando Bittemcourt, Martin Nakouzi, Ilaria Pola, Matthew Wong, Przemyslaw Radoslaw Kac, Ana Paula Bernardes Real, Chloe Witherow, Thomas K Karikari, Alexis Moscoso, Michael Schöll, Andrea L. Benedet, Tharick A Pascoal, Nicholas J. Ashton, Eduardo R. Zimmer, Henrik Zetterberg, Kaj Blennow, Pedro Rosa‐Neto

**Affiliations:** ^1^ Universidade Federal do Rio Grande do Sul, Porto Alegre, Rio Grande do Sul, Brazil; ^2^ McGill University, Montreal, QC, Canada; ^3^ University of Aarhaus, Aarhaus, Denmark; ^4^ University of Gothenburg, Gothenburg, Sweden; ^5^ University of Gothenburg, Mölndal, Sweden; ^6^ Nuclear medicine department and Molecular Imaging Group, Instituto de Investigación Sanitaria de Santiago de Compostela, Santiago de Compostela, Galicia, Spain; ^7^ Department of Psychiatry and Neurochemistry, Institute of Neuroscience and Physiology, Sahlgrenska Academy, University of Gothenburg, Gothenburg, Sweden; ^8^ Department of Psychiatry and Neurochemistry, Institute of Neuroscience and Physiology, The Sahlgrenska Academy, University of Gothenburg, Mölndal, Sweden; ^9^ University of Pittsburgh, Pittsburgh, PA, USA; ^10^ Banner Alzheimer's Institute, Phoenix, AZ, USA; ^11^ UFRGS, Porto Alegre, Brazil

## Abstract

**Background:**

Alzheimer's disease (AD) plasma biomarkers have transformed the field due to their scalability and minimal invasiveness, especially when evidence of AD biomarker abnormality is required for novel anti‐amyloid immunotherapies. Among plasma biomarker candidates, phosphorylated tau (*p*‐tau) variants show most promise for clinical application. While establishing cutoffs is crucial for implementation, understanding continuous‐level changes is critical for accurate interpretation in research and clinical settings. Given the variety of *p*‐tau biomarkers and emerging assays, robust estimates of continuous‐level plasma *p*‐tau changes across the clinical spectrum of AD are needed.

**Method:**

We screened databases for studies (01‐Jul‐1984 to 09‐Dec‐2024) reporting plasma *p*‐tau concentrations alongside Aβ‐PET, CSF biomarkers, or neuropathology. Plasma *p*‐tau fold‐changes across the AD clinical spectrum were assessed in two analyses: i) from CU Aβ‐ to CU Aβ+, MCI Aβ+, and Aβ+ dementia and (ii) CI Aβ‐positive vs. CI Aβ‐negative. Random‐effects models using the ratio of means method estimated fold‐change effect sizes. This PRISMA‐compliant study was pre‐registered (PROSPERO‐ID: CRD42023422143).

**Result:**

Of 2112 titles screened, 71 studies were included in this preliminary analysis. Plasma *p*‐tau217 showed a stepwise increase across the AD clinical spectrum, increasing 2.17‐fold (95%CI 1.98–2.37) in CU Aβ‐positive (*n* = 1451), 3.16‐fold (95%CI 2.84–3.52) in MCI Aβ‐positive (*n* = 1185), and 4.95‐fold (95%CI 4.18–5.87) in Aβ‐positive dementia (*n* = 925) compared with CU Aβ‐negative (*n* = 3973) (Figure 1). When comparing Aβ‐positive CI vs. Aβ‐negative CI individuals, *p*‐tau217 increased nearly twice as much (3.58‐fold, 95%CI 3.25–3.93; *n* = 5995) compared to *p*‐tau181 (1.71‐fold, 95%CI 1.61–1.82; *n* = 8342) and *p*‐tau231 (1.71‐fold, 95%CI 1.53–1.90; *n* = 1019) (Figure 2), and similar to *p*‐tau217/np‐tau217 (%p‐tau217: 3.59‐fold, 95%CI 2.74‐4.71; *n* = 1019). Effect size summaries for each *p*‐tau variant across analyses are shown in Figure 3. Sensitivity analyses preserving only the median‐performing assay in head‐to‐head studies led to similar results.

**Conclusion:**

P ‐tau217 is the plasma biomarker with the largest dynamic range across the entire clinical AD spectrum, with its large AD‐related effect sizes in symptomatic patients making it a robust candidate for clinical implementation. Results do not demonstrate any consistent superiority of %p‐tau217 over *p*‐tau217, but few studies used the ratio. Data extraction is ongoing until March/2025. A paired diagnostic accuracy meta‐analysis was also submitted.